# Moral Foundations and Voting Intention in Italy

**DOI:** 10.5964/ejop.v13i4.1391

**Published:** 2017-11-30

**Authors:** Patrizia Milesi

**Affiliations:** a Department of Psychology, Catholic University of the Sacred Heart, Milan, Italy; Department of Psychology, Webster University Geneva, Geneva, Switzerland; London School of Economics, London, United Kingdom

**Keywords:** moral foundations, vote, ideological orientation, morality, politics

## Abstract

Based on the view of morality proposed by the Moral Foundations Theory, this paper investigates whether voting intention is associated with moral foundation endorsement in not perfectly bipolar electoral contexts. Three studies carried out in Italy from 2010 to 2013, showed that controlling for ideological orientation, moral foundation endorsement is associated with voting intention. In Study 1 and 3, in fictitious and real national elections, intention to vote for right-wing political groups rather than for left-wing rivals was associated with Sanctity, confirming previous results obtained in the U.S. Furthermore, as a function of the specific competing political groups in each of the examined contexts other moral foundations predicted voting intention. In Study 1, Care and Authority predicted voting intention for the major political groups rather than for an autonomist party that aimed at decreasing central government’s fiscal power in favor of fiscal regional autonomy. In Study 3, Loyalty predicted the intention to vote for the major parliamentarian parties rather than for a movement that aimed at capturing disaffection towards traditional politics. In Study 2, at real regional elections, Loyalty predicted voting intention for the incumbent right-wing governor rather than for the challengers and Fairness predicted voting intention for left-wing extra-parliamentarian political groups rather than for the major left-wing party. Thus multiple moral concerns can be associated with voting intention. In fragmented and unstable electoral contexts, at each election the context of the competing political groups may elicit specific moral concerns that can contribute to affect voting intention beyond ideological orientation.

In recent years, increasing attention has been given to moral concerns as an individual motivation to vote (or not vote). Voters are said to vote for their vision of a good society, that is, what the moral priorities of a society should be, and how they should be achieved (e.g., Lakoff, 2002). When attitudes towards candidates and political issues are perceived as moral convictions, that is as being strong, absolute, and non-arbitrary judgments that reflect personal core moral beliefs, they become especially compelling and they increase both intention to vote and real voter turnout (Morgan, Skitka, & Wisneski, 2010; Skitka & Bauman, 2008).

This paper investigates the relationship between moral concerns and voting by making use of the pluralistic view of morality developed by the Moral Foundations Theory. Based on a broad conceptualization of morality, the Moral Foundations Theory (see Graham et al., 2013 for a review; Haidt, 2001) proposes five innate universal moral concerns, or moral foundations, upon which people decide whether something is morally right or wrong. Moral foundations have been shown to be related to political ideology as well as to a host of political attitudes (e.g., Graham, Haidt, & Nosek, 2009; Graham et al., 2011; Koleva, Graham, Iyer, Ditto, & Haidt, 2012) and judgments about candidates (Iyer, Graham, Koleva, Ditto, & Haidt, 2010). The association between moral foundation endorsement and voting has received comparatively more recent attention and limited to U.S. electoral contexts. Johnson and colleagues (Johnson et al., 2014) showed that, at the 2008 and 2012 U.S. presidential elections, *low* endorsement of moral foundations was associated with non-voting behavior. Franks and Scherr (2015) found that, at the 2012 U.S. presidential elections, moral foundation endorsement was associated with voting choice beyond relevant socio-demographic variables.

Thus vote seems to be motivated by voters’ moral concerns. Given that moral concerns are associated with ideological orientation (Graham et al., 2009) and ideological orientation itself is associated with vote (e.g., Freire, 2006; Van der Eijk, Schmitt, & Binder, 2005), the association between moral concerns and vote might be partly accounted for by the individual’s ideological orientation. In this paper, we argue that, although ideological orientation is a strong predictor of vote, it does not capture all the moral concerns that can underlie voting; rather, depending on the features of each electoral context, specific moral concerns would retain a unique predictive power on vote choice. In the following sections, first we briefly review the literature on moral foundations and their association with ideological orientation and vote. Then, we present three studies where we examine the association between moral foundations and voting intention taking into account the individual’s ideological orientation.

## Moral Foundations, Ideological Orientation, and Vote

The Moral Foundations Theory has highlighted a set of five basic universal moral concerns that guide judgments of right and wrong (e.g., Graham et al., 2013): 1) the Care/Harm foundation that deals with the protection and nurture of those who are vulnerable, suffering or in need; 2) the Fairness/Cheating foundation that is concerned with the relevance of cooperation and of trustworthy and fair relationships; 3) the Loyalty/Betrayal foundation that has evolved from the need to build and maintain coalitions and is focused on the value of being loyal to one’s group, team or a coalition; 4) the Authority/Subversion foundation that deals with obedience to hierarchies and authorities and respect for established social institutions; and 5) the Sanctity/Degradation foundation, that includes concerns for the safeguard of physical and spiritual purity and emphasizes the sacredness of life.

The Care and Fairness foundations focus on how to protect individuals and on how to teach them to respect each others’ rights. Accordingly, they are called “individualizing foundations.” Care and Fairness concerns are especially likely to be activated by issues related to an equitable balance of power and distribution of resources, and they are positively associated with preferences for social relations being equal rather than hierarchical. The Loyalty, Authority, and Sanctity foundations aim at binding individuals into roles and duties in order to strengthen groups and institutions. Accordingly, they are called “binding foundations.” Loyalty, Authority and Sanctity concerns are especially likely to be activated by issues related to the protection of traditional communal norms and identities, and they are positively associated with preferences for conformity rather than openness to change (Federico, Weber, Ergun, & Hunt, 2013; Graham et al., 2009).

Moral foundation endorsement is related to ideological orientation. Early studies showed that conservatives, or people who self-place on the political right, emphasize all the five moral foundations to equal extents, while liberals, or people who self-place on the political left, are likely to give more weight to Care and Fairness foundations than to the binding foundations (Graham et al., 2009; see also Graham, Nosek, & Haidt, 2012). More recent studies argue that the association between moral foundations and ideological orientation is likely to be more complex than initially thought. Particular constellations of moral foundation endorsement are associated with political self-definitions that the liberal-conservative divide, or the left-right continuum, cannot capture, for example the self-definition as “libertarian” (Iyer, Koleva, Graham, Ditto, & Haidt, 2012). Moreover, individuals who self-define ideologically in a similar way, e.g. as liberals or as conservatives, may show distinct constellations of moral concerns. In this regard, based on a view of ideology as multifaceted (Feldman & Johnston, 2014), Weber and Federico (2013) highlighted six different subtypes of liberal and conservatives. Each subtype showed a distinct pattern of moral foundation endorsement. As a result, a given moral foundation could be endorsed to different degrees by people who share similar ideological orientation. For example, individuals who were slightly left of centre on fiscal issues but endorsed very liberal positions on all social issues except for immigration and abortion, the so-called inconsistent liberals, endorsed Sanctity concerns more strongly than the consistent liberals, who took liberal positions on both economic and social issues. Similarly, individuals who were relatively moderate on fiscal issues but very conservative on social issues, i.e. the so-called social conservatives, also endorsed Sanctity concerns more strongly than the consistent conservatives who took conservative positions on both economic and social issues.

In the U.S., moral foundation endorsement has turned out to be associated also with electoral behavior, or voting intentions. Across four studies carried out during the 2008 and 2012 U.S. elections, Johnson and colleagues (2014) showed that low endorsement of moral foundations predicts non-voting: people who self-defined as liberal, or conservative, but scored low on endorsement of the moral foundations that are stereotypically associated with their self-identified political group, were more likely to have not voted in the past and to intend not to vote in the future. The mismatch between one’s political group and one’s own individual moral profile probably weakened the motivation to support either party during the elections. These results suggest that high endorsement of moral foundations that are stereotypically associated with a political group, may increase the intention to vote for the candidate of that group. They also imply that ideological self-definition may be not sufficient per se for people intending to vote, but it needs to be combined with relevant moral concerns. At the 2012 U.S. presidential elections, Franks and Scherr (2015) carried out three studies to investigate whether moral foundation endorsement is associated with vote choice by examining voting intention as well as retrospective self-reported voting behavior. The most reliable unique foundation predictor of candidate choice was Sanctity, which led participants to choose the Republican Romney rather than the Democratic Obama.

Although Franks and Scherr controlled for the effects of gender, income, ethnicity, and religiosity, they did not control for the effect of ideological orientation. Given that ideological orientation is associated with both moral foundation endorsement (Graham et al., 2009) and vote (Freire, 2006; Van der Eijk, Schmitt, & Binder, 2005), those results leave open the question as to whether ideological orientation accounts for the association between moral foundations and vote choice. Our basic idea is that the comparative context in which voters have to make their choice, that is, the voting alternatives represented by the competing political groups, could activate distinct moral concerns that would not be completely captured by the individual’s ideological orientation. In this regard, Iyer and colleagues (2010) observed that distinct moral foundations can be associated with judgments about different candidates although they share similar ideological orientation and similar policy positions. Actually, in the U.S. 2008 democratic primary elections, both candidates belonged to the Democratic Party and all the participants were self-identified Democrats thus probably prioritizing the individualizing foundations over the binding ones (Graham et al., 2009). While ideological orientation did not predict any favorability judgments about the competing candidates, Fairness endorsement predicted favor for Obama over Clinton, while Loyalty and Authority endorsement predicted favor for Clinton over Obama.

## Aim

**Figure 1:**
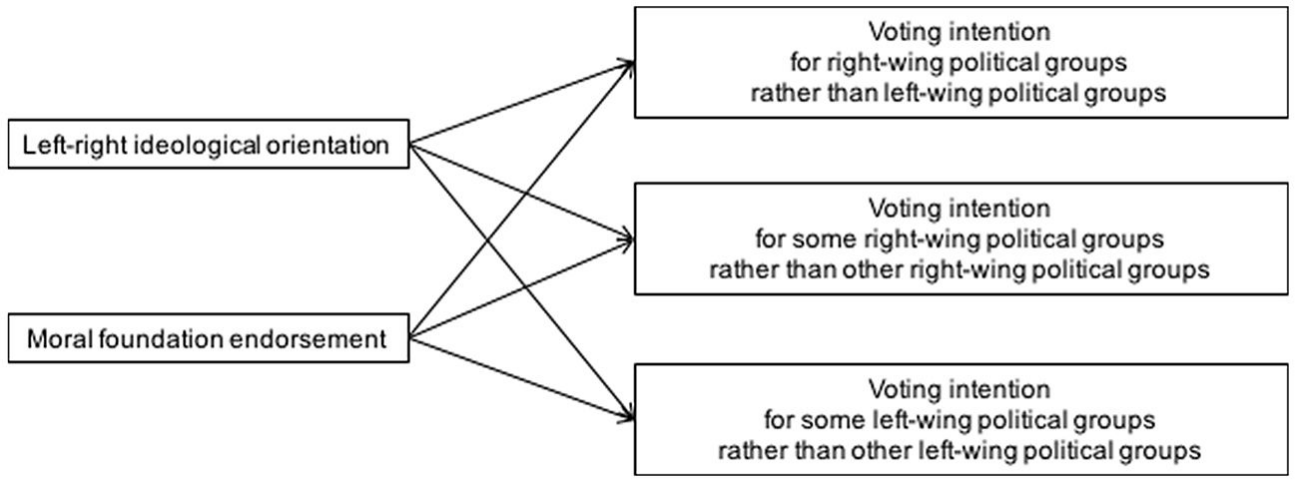
Conceptual diagram of the association between ideological orientation, moral foundation endorsement, and voting intention.

The present research aims to investigate the relationship between moral foundation endorsement and voting intention, controlling for respondents’ ideological orientation, in electoral contexts where the competition involves more than two political groups, some of which located at the opposite sides of the left-right continuum, some others at the same side. Figure 1 visualizes the investigated associations between ideological orientation, moral foundation endorsement, and voting intention in such electoral contexts.

Our basic idea is that, although ideological orientation is the strongest predictor of voting intention, in each electoral context distinct moral foundations would retain a unique predictive power of voting intention. Previous research supports this view. First, ideological orientation does not predict judgment about candidates who are located on the same side of the left-right spectrum and share similar policy positions; however, moral foundation endorsement does predict it. Iyer and colleagues (2010) showed that ideological orientation did not correlate with favorability for Clinton over Obama, or vice versa, at the 2008 Democratic primaries, where only Democratic candidates were competing, while some moral foundations did. Second, the relationship between moral foundations and ideological orientation is more complex than initially described. Weber and Federico (2013) not only showed that different subtypes of liberals and conservatives displayed distinct profiles of moral foundation endorsement, but also that individuals who are self-placed in similar positions along the left-right spectrum can give different weight to the same moral concerns. For example, consistent liberals and consistent conservatives placed less emphasis on Sanctity concerns than, respectively, inconsistent liberals and social conservatives. That research suggests that specific moral concerns can be associated with voting intention in a way that is not completely captured by the individual’s ideological orientation.

The present research was carried out in Italy between 2010 and 2013. In the examined electoral contexts, the choice always involved a main right-wing political group and a main rival left-wing group; in this, they can be considered as partly similar to the U.S. bipolar electoral contexts. As described above, at the 2012 U.S. presidential elections, the most reliable unique foundation predictor of candidate choice was Sanctity, which drove participants to support the conservative rather than the democratic candidate (Franks & Scherr, 2015). Firstly, we expected that the individuals’ ideological orientation would be the strongest predictor of their voting intention. However, we also expected that, across studies, Sanctity concerns would be associated with the intention to vote for the main right-wing political group rather than for its main left-wing rival, beyond the effect of respondents’ ideological orientation. Finally, we expected that the intention to support a political group in a given electoral context might be imbued also with other moral concerns than Sanctity. Which moral foundation is associated with voting intention would depend on the comparative context where participants make their choice because different competing political groups and their distinct programs would elicit different moral concerns. For more detailed explanations see the individual studies.

## Study 1

When Study 1 was carried out, there were three main political groups in the Italian Parliament. The major right-wing party, Popolo della Libertà, was in government. This party supported typical right-wing policies in favor of free market economy, law and order, and traditional family values. The parliamentary opposition was led by the major left-wing party, Partito Democratico: this party supported typical left-wing policies that favored equal opportunities, civil liberties, and tolerance for diversity (Marangoni & Verzichelli, 2015; Massetti & Toubeau, 2013). This led us to expect that, similarly to previous studies carried in the U.S., voting intention for the major right-wing party rather than for the major left-wing one would be associated with Sanctity concerns. The third political group in Parliament was an autonomist party, Lega Nord, which was the main ally of the major right-wing party. This party aimed at decreasing central government’s fiscal power in favor of regional fiscal autonomy. If regional fiscal autonomy had been implemented as this party wanted, this would have advantaged the richer regions while leaving the poorer ones without basic public services, and it would have deprived the central government of much of its power. We reasoned that those requests could raise both concerns for care of vulnerable people and concerns for the respect of established institutions like central state, i.e. Care and Authority concerns.

Participants were asked to indicate which of these three political groups they would vote for if there had been national elections.

### Method

#### Participants

In 2010, 319 Italian students were contacted during classes. Only participants who answered the Moral Foundations Questionnaire and stated their ideological orientation along the left-right continuum as well as their voting intention were included in the analyses. This left 216 participants (67.7% of those who were initially contacted; 176 women, age *M* = 21.12 years, *SD* = 4.15 years; political interest *M* = 3.74, *SD* = 1.62 on a 7-point scale from 1 = *not interested at all* to 7 = *very interested*; religious attendance *M* = 3.09, *SD* = 1.64 on a 5-point scale from 1 = *never* to 5 = *once a week*).

#### Measures

##### Moral foundations

Participants completed the Italian version (Bobbio, Nencini, & Sarrica, 2011) of the Moral Foundations Questionnaire (MFQ30, July 2008; full version by Graham, Haidt, & Nosek, available at www.moralfoundations.org; Care α = .69; Fairness α = .66; Loyalty α = .52; Authority α = .59; Sanctity α = .74). For some scales, the reliability scores are not very high but they are close to those obtained in previous studies (e.g., Federico et al., 2013; Graham et al., 2009); this could be due to the MFQ’s aim of capturing the greatest scope of each foundation.

##### Ideological orientation

Participants were asked to self-locate along the left-right continuum using a 5-point scale, ranging from 1 = *left-wing oriented* to 5 = *right-wing oriented*. The scale also included an answer option to exclude those who refused to self-identify along the left-right continuum (*I do not self-define by any one of these labels*). Participants who did not self-define along the left-right continuum were excluded from the analyses. Eighty participants self-defined as left- or centre-left; 19 as centre; 117 as right- or centre-right.

##### Voting intention

Participants were asked to state their voting intention as if they were to vote at the national elections the next day. One hundred and one participants stated they would vote for the incumbent right-wing party, Popolo della Libertà; 78 participants for the opposition left-wing party, Partito Democratico; 37 participants for the autonomist right-wing party, Lega Nord.

### Results

First, we carried out bivariate correlations between participants’ moral foundation endorsement, their ideological orientation, and their voting intention. In the bivariate correlations, voting intention was coded to distinguish between voting intention for the incumbent right-wing party Popolo della Libertà, coded as 1, and voting intention for the opposition left-wing party Partito Democratico, coded as 0.

As shown in Table 1, the correlations between moral foundation endorsement and ideological orientation were fairly consistent with previous findings regarding the association between moral foundation endorsement and ideology (e.g., Graham et al., 2009).

**Table 1:** Bivariate Correlations Between Moral Foundation Endorsement, Ideological Orientation, and Voting Intention for the Incumbent Right-Wing Party Popolo della Libertà vs. the Opposition Left-Wing Party Partito Democratico in 2010, Study 1 (N = 179)

Variable	1	2	3	4	5	6	7
1. Care	-						
2. Fairness	.60***	-					
3. Loyalty	.34***	.28***	-				
4. Authority	.13	.11	.65***	-			
5. Sanctity	.34***	.26***	.55***	.56***	-		
6. Ideological orientation	-.14^†^	-.21**	.14^†^	.33***	.22**	-	
7. Voting intention	-.13	-.11	.15*	.29***	.28***	.79***	-

*Note.* Ideological orientation: from 1 = left-wing oriented to 5 = right-wing oriented. Voting intention coded as 0 = intention to vote for the opposition left-wing party Partito Democratico; 1 = intention to vote for the incumbent right-wing party Popolo della Libertà.

^†^*p* < .06. *p < .05. **p < .01. ***p < .001.

Being right-wing oriented also correlated positively with the intention to vote for the incumbent right-wing rather than for the opposition left-wing party. More interestingly, both Authority and Sanctity endorsement were positively associated with participants’ intention to vote for the incumbent right-wing rather than for the opposition left-wing party.

Then, we carried out a nominal regression of voting intention on ideological orientation and socio-demographic variables, i.e., gender, age, religious attendance, and political interest. Voting intention was treated as a nominal variable with three levels: voting for the incumbent right-wing party, voting for the autonomist right-wing party, and voting for the opposition left-wing party. Voting for the left-wing party served as the reference group. Only ideological orientation significantly predicted participants’ voting intention. The further participants leaned towards right-wing, the greater they intended to vote for the incumbent right-wing party (*B* = 2.103, *SE* = 0.322, Wald = 42.573; *OR* = 8.190, 95% CI[4.354, 15.403]; *p* < .001) and the autonomist right-wing party (*B* = 3.505, *SE* = 0.534, Wald = 43.082; *OR* = 33.281, 95% CI[11.686, 94.782]; *p* < .001) rather than for the left-wing one.

In order to investigate whether moral foundation endorsement would predict participants’ voting choice among the incumbent right-wing party, the autonomist right-wing party, and the opposition left-wing party, we ran a second nominal regression including as predictors the five moral foundations and, once again, ideological orientation. As shown in Table 2, ideological orientation was the strongest predictor of participants’ voting intention. Consistent with our expectation, however, also Sanctity endorsement emerged as a unique predictor of voting intention for the incumbent right-wing rather than for the left-wing party. Moreover, endorsement of Care and Authority uniquely predicted participants’ intention to vote for the left-wing rather than for the autonomist right-wing party.

**Table 2:** Nominal Regression of Voting Intention in 2010 on Moral Foundation Endorsement and Ideological Orientation, Study 1 (N = 216)

Predictors	*B*	*SE*	OR	95% CI	Wald	*p*
Voting intention for the incumbent right-wing party, Popolo della Libertà						
Care	-0.787	0.500	0.455	0.171, 1.214	2.473	.116
Fairness	0.543	0.630	1.722	0.501, 5.917	0.744	.388
Loyalty	0.043	0.568	1.044	0.343, 3.180	0.006	.940
Authority	-0.427	0.533	0.652	0.230, 1.853	0.643	.423
Sanctity	0.888	0.397	2.430	1.116, 5.291	4.999	.025
Ideological orientation	2.069	0.311	7.916	4.302, 14.568	44.206	< .001
Voting intention for the autonomist right-wing party, Lega Nord						
Care	-1.556	0.629	0.211	0.061, 0.724	6.115	.013
Fairness	1.082	0.746	2.951	0.684, 12.734	2.105	.147
Loyalty	0.907	0.737	2.478	0.584, 10.514	1.514	.218
Authority	-1.352	0.671	0.259	0.070, 0.963	4.064	.044
Sanctity	0.609	0.520	1.839	0.664, 5.094	1.372	.241
Ideological orientation	3.654	0.547	38.623	13.213, 112.899	44.576	< .001

*Note*. Voting intention for the opposition left-wing party, Partito Democratico, was the reference group. Ideological orientation: from 1 = left-wing oriented to 5 = right-wing oriented.

Another nominal regression, where voting intention for the autonomist right-wing party served as the reference group, showed that, always controlling for ideological orientation, endorsement of Care (*B* = 0.770, *SE* = 0.395; Wald = 3.791; *OR* = 2.159, 95% CI[0.995, 4.685]; *p* = .052) and endorsement of Authority (*B* = 0.925, *SE* = 0.423; Wald = 4.776; *OR* = 2.521, 95% CI[1.100, 5.778]; *p* = .029) also predicted the intention to vote for the incumbent right-wing rather than for the autonomist right-wing party.

**Figure 2:**
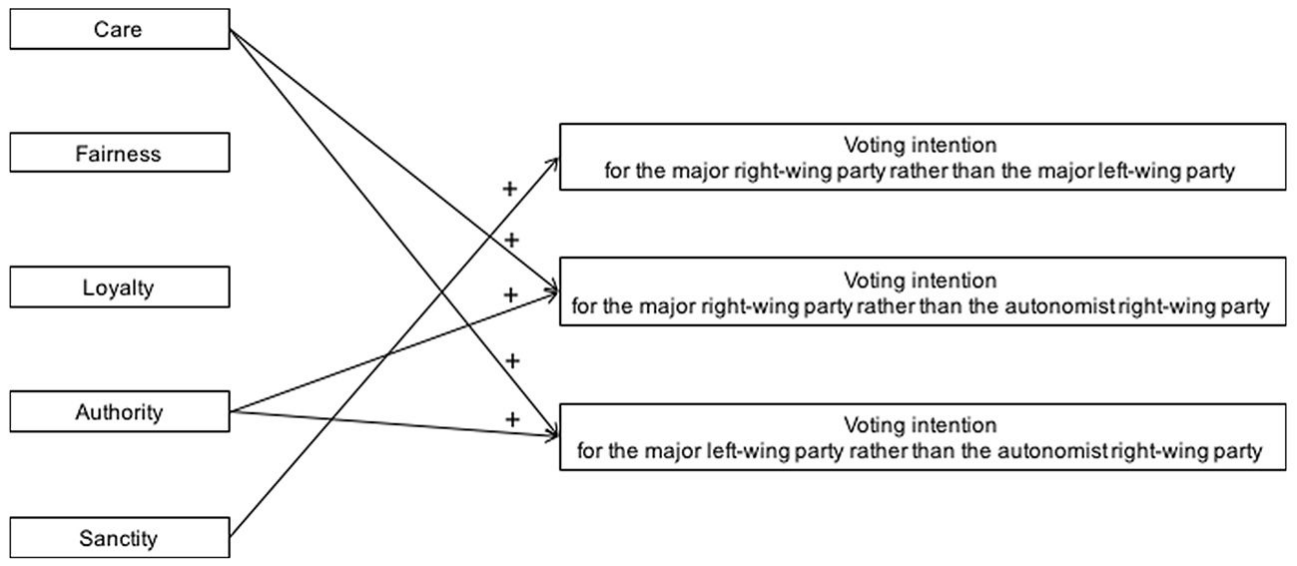
The association between distinct moral foundations and voting intention in fictitious national elections, Study 1. Visualization of the main results obtained in regression analyses, controlling for ideological orientation.

Figure 2 visualizes the association between moral foundations and voting intention, controlling for ideological orientation, as it emerged in Study 1 where the two major right-wing and left-wing parties and the autonomist right-wing party were competing in fictitious national elections.

### Discussion

Consistent with the literature on ideology and vote (e.g., Freire, 2006; Van der Eijk et al., 2005), the strongest predictor of voting intention turned out to be participants’ ideological self-definition. However, obtained results showed that also Sanctity concerns were associated with the intention to vote for the incumbent right-wing rather than for the opposition left-wing party. This result confirms our general expectation that Sanctity endorsement would predict voting intention for the main right-wing political groups rather than for the main left-wing competitors beyond participants’ ideological orientation. Moreover, as expected, distinct moral concerns were associated with voting intention for the right-wing autonomist party. Actually, Care and Authority concerns predicted support both for the right-wing Popolo della Libertà and for the left-wing Partito Democratico rather than for the autonomist right-wing Lega Nord. The emphasis that Lega Nord placed on regional fiscal autonomy might have raised concerns in supporters of other parties for the care of inhabitants of poorer regions, who would be deprived of basic public services if Lega Nord’s autonomist plans had been implemented, as well as concerns for the diminished authority of the central State.

Study 1 examined hypothetical elections. One could ask whether similar results would emerge in real elections, as in real-life elections electoral campaigns might raise the accessibility of both ideological orientation and moral concerns.

## Study 2

Study 2 examined the 2010 Lombardy regional elections. In 1999, institutional reforms introduced the direct election of regional presidents. Although the direct election of regional presidents can provide a chance for national parties to increase their strength at sub-national levels through support for *their* presidential candidates, it is very often the case that regional powerbrokers who can rely on widespread local networks take advantage of regional elections to further strengthen their territorial power and get to challenge national party elites (Wilson, 2015). As a result, a territorial logic often governs Italian regional elections in a way that is quite independent from national dynamics (Passarelli, 2013). In the 2010 Lombardy regional elections, the incumbent right-wing candidate (Roberto Formigoni) had been governing the region for fifteen years and had built a solid territorial powerbase by establishing strong links with regional economic, health, and educational institutions. The major left-wing challenger (Filippo Penati) was a candidate explicitly supported by the national left-wing party. A range of other minor left-wing challengers represented extra-parliamentary political groups (e.g., a labor unionist, a representative of the no-global movement) and ran independently from the candidate that the national left-wing party supported. In such a context, Sanctity concerns might be associated with the intention to vote for the main right-wing candidate rather than for the left-wing challenger (Franks & Scherr, 2015). However, the territorial logic that governs regional elections could also raise moral concerns about the importance of being loyal to one’s in-group and promoting its interests.

### Method

#### Participants

In March 2010, one month before the regional elections, 302 Italians who lived in Lombardy were approached in public spaces and asked to complete a short questionnaire. Only participants who answered all the questions about their moral foundation endorsement, self-defined along the left-right continuum, and reported their voting intention at the upcoming elections were included in the analyses. One hundred and sixty-four participants constituted the final sample (54.3% of those initially involved; 94 women, age range = 18 – 72 years, age *M* = 40.42, *SD* = 13.90 years). Fifteen participants had compulsory education, 77 had secondary education, 71 had tertiary education, and one participant did not answer the question about educational level.

#### Measures

##### Moral foundations

As in Study 1, moral foundation endorsement was measured through the MFQ30 (Care α = .66; Fairness α = .61; Loyalty α = .80; Authority α = .82; Sanctity α = .71).

##### Ideological orientation

Participants stated their ideological orientation along the same scale used in Study 1. Participants who refused to self-define along the left-right continuum were excluded from the analyses. Seventy-two participants self-defined as left or centre-left; twenty as centre; 72 as right or centre-right.

##### Voting intention

Participants were asked which candidate they intended to vote for at the upcoming regional elections. Sixty-one participants answered that they would support the right-wing candidate (Formigoni), 44 answered that they would vote for the major left-wing challenger (Penati) and 59 said that they would vote for one of the independent left-wing challengers.

### Results

As in Study 1, first we carried out bivariate correlations between participants’ moral foundation endorsement, their ideological orientation, and voting intention. Voting intention distinguished between the intention to vote for the incumbent right-wing candidate Formigoni, coded as 1, and the intention to vote for the major left-wing challenger Penati, coded as 0 (Table 3).

**Table 3:** Bivariate Correlations Between Moral Foundation Endorsement, Ideological Orientation, and Voting Intention for the Incumbent Right-Wing Candidate (Formigoni) vs. the Major Left-Wing Challenger (Penati), Study 2 (N = 105)

Variable	1	2	3	4	5	6	7
1. Care	-						
2. Fairness	.54***	-					
3. Loyalty	-.02	-.13	-				
4. Authority	-.08	-.11	.86***	-			
5. Sanctity	.31***	.09	.61***	.63***	-		
6. Ideological orientation	-.20*	-.22*	.57***	.63***	.44***	-	
7. Voting intention	-.22*	-.18^†^	.65***	.69***	.49***	.91***	-

*Note.* Ideological orientation: from 1 = left-wing oriented to 5 = right-wing oriented. Voting intention coded as 0 = intention to vote for the major left-wing challenger (Penati); 1 = intention to vote for the incumbent right-wing candidate (Formigoni).

^†^*p* < .06. *p < .05. **p < .01. ***p < .001.

As shown in Table 3, once again the pattern of correlations between moral foundation endorsement and ideological orientation was consistent with previous findings (Graham et al., 2009). Right-wing ideological orientation strongly correlated with the intention to support the incumbent right-wing candidate rather than the major left-wing challenger. More relevant to our purpose, Care endorsement was associated with the intention to vote for the major left-wing challenger rather than for the incumbent right-wing candidate. Loyalty, Authority, and Sanctity endorsement positively correlated with the intention to vote for the incumbent right-wing candidate rather than for the major left-wing challenger.

Similarly to Study 1, firstly, we performed a nominal regression of voting intention on ideological orientation and socio-demographic variables, i.e., gender, age, and educational level; no information was collected in Study 2 about participants’ religious attendance or their political interest. Voting intention was treated as a nominal variable on three levels: voting for the incumbent right-wing candidate, voting for the major left-wing challenger, and voting for one of the independent left-wing candidates. Voting for the major left-wing challenger served as the reference group. Once again, ideological orientation was a significant predictor of participants’ voting intention and no other significant predictors emerged. Right-wing orientation predicted voting intention for the incumbent right-wing candidate (*B* = 2.325, *SE* = 0.374, Wald = 38.556; *OR* = 10.230, 95% CI[4.910, 21.313]; *p* < .001) as well as for the independent left-wing challengers (*B* = 0.546, *SE* = 0.225, Wald = 5.859; *OR* = 1.726, 95% CI[1.109, 2.684]; *p* = .015) rather than for the major left-wing challenger.

To examine whether moral foundation endorsement would predict participants’ voting choice among the incumbent right-wing candidate, the major left-wing challenger, and the other independent left-wing challengers, a nominal regression was carried out, with the five moral foundations and the ideological orientation as predictors (Table 4).

**Table 4:** Nominal Regression of Voting Intention at the 2010 Lombardy Regional Elections on Moral Foundation Endorsement and Ideological Orientation, Study 2 (N = 164)

Predictors	*B*	*SE*	OR	95% CI	Wald	*p*
Voting intention for the incumbent right-wing candidate (Formigoni)						
Care	-0.514	0.469	0.598	0.238, 1.499	1.202	.273
Fairness	0.483	0.511	1.621	0.596, 4.410	0.894	.344
Loyalty	0.585	0.298	1.795	1.001, 3.217	3.858	.050
Authority	0.230	0.337	1.259	0.651, 2.435	0.467	.494
Sanctity	0.410	0.340	1.506	0.773, 2.935	1.450	.228
Ideological orientation	2.241	0.403	9.406	4.269, 20.726	30.923	< .001
Voting intention for the independent left-wing challengers						
Care	-0.250	0.328	0.779	0.409, 1.481	0.582	.446
Fairness	0.915	0.376	2.497	1.195, 5.219	5.919	.015
Loyalty	0.123	0.179	1.131	0.797, 1.605	0.476	.490
Authority	-0.028	0.241	0.972	0.606, 1.560	0.014	.907
Sanctity	0.146	0.243	1.157	0.718, 1.862	0.359	.549
Ideological orientation	0.691	0.248	1.995	1.227, 3.244	7.762	.005

*Note*. Voting intention for the major left-wing challenger (Penati) was the reference group. Ideological orientation: from 1 = left-wing oriented to 5 = right-wing oriented.

As displayed in Table 4, ideological orientation was the strongest predictor of participants’ voting intention. Loyalty endorsement also emerged as a significant predictor of voting intention for the incumbent right-wing candidate rather than for the major left-wing challenger. Moreover, Fairness endorsement was a unique predictor of voting intention for one of the independent left-wing challengers rather than for the major left-wing challenger.

In another nominal regression, where voting intention for one of the independent left-wing challengers served as the reference group, always controlling for ideological orientation, Loyalty endorsement also marginally predicted voting intention for the incumbent right-wing candidate rather than for one of the independent left-wing challengers (*B* = 0.462, *SE* = 0.266, Wald = 3.014; *OR* = 1.587, 95% CI[0.942, 2.672]; *p* = .083).

**Figure 3:**
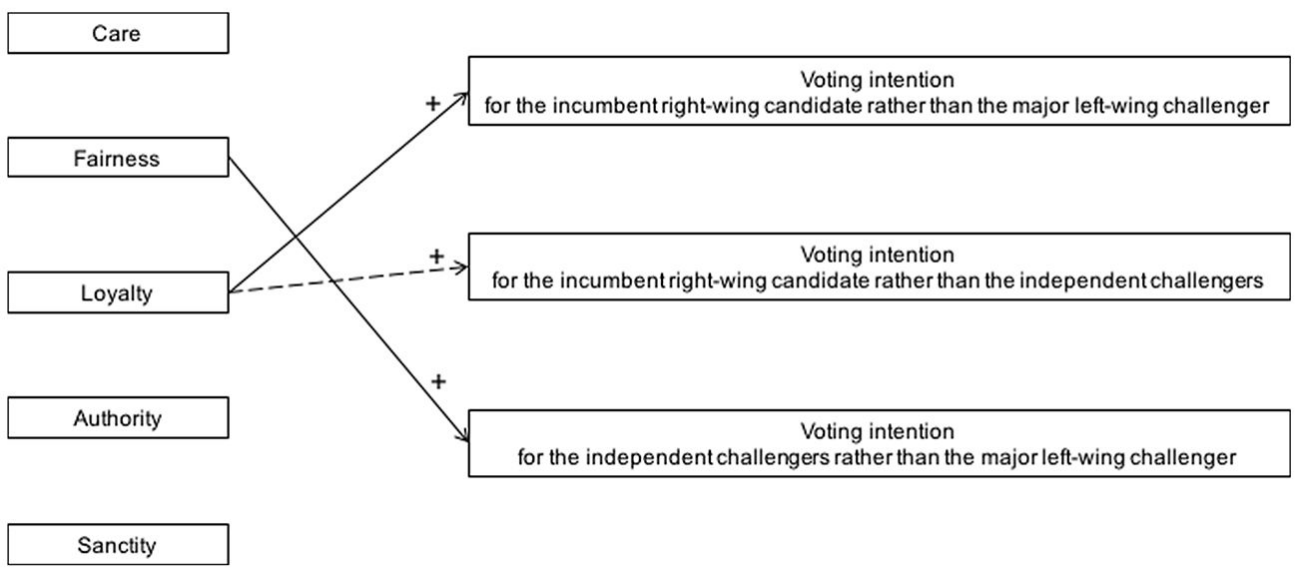
The association between distinct moral foundations and voting intention in 2010 Lombardy regional elections, Study 2. Visualization of the main results obtained in regression analyses, controlling for ideological orientation. The dashed arrow represents a marginally significant association.

Figure 3 visualizes the association between moral foundations and voting intention, controlling for ideological orientation, as it emerged in Study 2 where the two major right-wing and left-wing candidates and other minor independent left-wing challengers were competing in regional elections.

### Discussion

Study 2 shows that, although the individual’s ideological orientation was the strongest predictor, specific moral concerns were associated with voting intention in 2010 Lombardy regional elections. Differently from most studies where Sanctity concerns were associated with the intention to support the major right-wing candidate rather than the major left-wing one, in Study 2 participants’ voting intention for the incumbent right-wing candidate rather than for the left-wing challengers turned out to be associated mainly with their concerns for being loyal to their in-group. Thus, beyond participants’ ideological orientation, it was their moral concern for safeguarding and promoting their in-group’s interests that led them to support the incumbent right-wing candidate. This result is consistent with the territorial logic that often governs regional elections in Italy (Passarelli, 2013) and it may have been due to the candidate’s long regional presidency and to his strong influence on the financing of regional institutions.

The results of Study 2 also suggest that, in the 2010 Lombardy regional elections, participants who intended to vote for one of the representatives of extra-parliamentary left-wing groups rather than for the candidate the national left-wing party supported probably responded to a basic moral concern for justice that went beyond their ideological orientation, as well as beyond the requests advanced by the national party.

## Study 3

Study 3 was carried out during the month before the 2013 Italian national elections. The context of the 2013 national elections was peculiar. The government formed by the right-wing party that had won previous national elections collapsed in November 2011. Instead of calling for national elections, thanks to a special mandate of the Republic President, the former EU commissioner Mario Monti formed a new technocratic government that was supported by a grand coalition between the two major right- and left-wing political groups. Despite this, at the end of 2012, the technocratic government was forced to resign and this led to the 2013 national elections. Within such a complex context, citizens’ ideologies became weaker in influencing their voting choice, their political disengagement grew, and their propensity not to vote for traditional parties increased. As a consequence, before the 2013 national elections the percentage of undecided or reticent voters was exceptionally high (Vegetti, Poletti, & Segatti, 2014). At the 2013 national elections, Italian voters could choose between four political groups. On the one hand, there were two traditional political coalitions, that is, the main right-wing coalition and the main left-wing coalition: this led us to expect that, consistent with previous studies, Sanctity concerns would be associated with intention to vote for the right-wing coalition rather than for the left-wing one. However, also two outsiders of traditional politics participated in the electoral competition: another right-wing coalition led by the incumbent Prime Minister Monti, Con Monti per l’Italia, and the populist Movimento Cinque Stelle spiritually led by political activist and comedian Beppe Grillo. The electoral list led by Monti entered the race only at the beginning of January 2013, less than two months before the election. On the contrary, the Movimento Cinque Stelle was born in late 2009: since regional elections in 2010, the movement had been very successful in capturing widespread disaffection and mistrust towards traditional political parties, and had attracted many disappointed voters from both the right-wing and the left-wing major political parties. Therefore, at the 2013 national elections, the movement represented an important voting alternative (Baldini, 2013; Donovan, 2015; Fella & Ruzza, 2013). While we had no specific expectations about the moral concerns raised by the Monti coalition since it was very recently founded and mainly technocratic in character, we expected that support for traditional right-wing and left-wing parties rather than for the Movimento Cinque Stelle could be associated either with concerns for loyalty towards one’s own political in-group or with concerns about the respect due to established institutions like traditional parties, i.e. either with Loyalty or with Authority concerns.

In the month before the Election Day, participants were asked to state which of these four political groups they would vote for at the upcoming elections.

### Method

#### Participants

Two hundred and ninety-one participants were invited to complete an online questionnaire through the Surveymonkey platform during the month before the 2013 Italian national elections. As in previous studies, only participants who answered the MFQ, self-defined along the left-right continuum, and stated their voting intention were retained in the analyses. Only 34.3% of those who were contacted responded to all these questions: the high dropout of participants is consistent with the unusually high percentage of undecided or reticent voters before the 2013 elections (Vegetti et al., 2014). The final sample thus comprised 100 participants (62 women; age range = 18 – 79, age *M* = 43.22, *SD* = 16.35 years; political interest *M* = 4.84, *SD* = 1.57 on a 7-point scale from 1 = *not interested at all* to 7 = *very interested*; religious attendance *M* = 3.09, *SD* = 1.68 on a 5-point scale from 1 = *never* to 5 = *once a week*). Seven participants had compulsory education, 38 had secondary education, and 55 had tertiary education.

#### Measures

##### Moral foundations

Participants completed the MFQ20 questionnaire, which is a shortened version of the MFQ30 questionnaire (www.moralfoundations.org), Care α = .66; Fairness α = .60; Loyalty α = .70; Authority α = .63; Sanctity α = .78).

##### Ideological orientation

Participants self-defined along the left-right continuum using the same scale used in previous studies. Participants who did not self-place along the left-right continuum were excluded from the analyses. Fifty-nine participants self-defined as left or centre-left, 13 centre, and 28 as right or centre-right.

##### Voting intention

Participants were asked to state their voting intention at the upcoming elections. Forty-one said they would vote for the left-wing coalition; 15 said they would vote for the challenging right-wing coalition; 23 said they would vote for the incumbent right-wing coalition led by Monti, Con Monti per l’Italia, and 21 said that they would vote for the Movimento Cinque Stelle.

### Results

Similarly to previous studies, first we ran bivariate correlations between participants’ moral foundation endorsement, their ideological orientation, and their voting intention. Voting intention was coded to distinguish between the intention to vote for the incumbent right-wing coalition (Con Monti per l’Italia, coded as 1), and the intention to vote for the challenging left-wing coalition (coded as 0).

As shown in Table 5, the association between moral foundation endorsement and ideological orientation is quite consistent with those of previous studies. Once again, right-wing orientation correlated positively with the intention to support the incumbent right-wing rather than the left-wing coalition. Moreover, Authority and Sanctity endorsement positively correlated with participants’ intention to vote for the incumbent right-wing rather than for the left-wing coalition.

**Table 5:** Bivariate Correlations Between Moral Foundation Endorsement, Ideological Orientation, and Voting Intention for the Incumbent Right-Wing Coalition, Con Monti per l’Italia, vs. the Challenging Left-Wing Coalition, Study 3 (N = 64)

Variable	1	2	3	4	5	6	7
1. Care	-						
2. Fairness	.49***	-					
3. Loyalty	.36**	.22	-				
4. Authority	.26*	.08	.59***	-			
5. Sanctity	.41***	.27*	.53***	.54***	-		
6. Ideological orientation	-.03	-.33**	.11	.34**	.29**	-	
7. Voting intention	.03	-.19	.12	.35**	.36**	.62***	-

*Note.* Ideological orientation: from 1 = left-wing oriented to 5 = right-wing oriented. Voting intention coded as 0 = intention to vote for the challenging left-wing coalition; 1 = intention to vote for the incumbent right-wing coalition, Con Monti per l’Italia.

*p < .05. **p < .01. ***p < .001.

Subsequently, we carried out a nominal regression of voting intention on ideological orientation and socio-demographic variables i.e., gender, age, educational level, religious attendance, and political interest. Voting intention was treated as a nominal variable with four levels: voting for the incumbent right-wing coalition, Con Monti per l’Italia, voting for the challenging right-wing coalition, voting for the left-wing coalition, and voting for the Movimento Cinque Stelle. Voting for the left-wing coalition served as the reference group. No socio-demographic variable emerged as significant predictor. Right-wing ideological orientation predicted intention to vote for the challenging right-wing coalition (*B* = 3.128, *SE* = 0.666, Wald = 22.049; *OR* = 22.835, 95% CI[6.188, 84.272]; *p* < .001), for the incumbent right-wing coalition (*B* = 1.325, *SE* = 0.397, Wald = 11.153; *OR* = 3.760, 95% CI[1.728, 8.181]; *p* = .001), and for the Movimento Cinque Stelle (*B* = 1.104, *SE* = 0.395, Wald = 7.832; *OR* = 3.017, 95% CI[1.392, 6.540]; *p* = .005) rather than for the left-wing coalition.

Finally, in order to investigate whether moral foundation endorsement would predict participants’ voting choice, we ran another nominal regression including participants’ moral foundation endorsement and, once again, their ideological orientation as predictors (Table 6).

**Table 6:** Nominal Regression of Voting Intention at the 2013 Italian National Elections on Moral Foundation Endorsement and Ideological Orientation, Study 3 (N = 100)

Predictors	*B*	*SE*	OR	95% CI	Wald	*p*
Voting intention for the incumbent right-wing coalition, Con Monti per l’Italia						
Care	0.093	0.698	1.097	0.280, 4.307	0.018	.894
Fairness	-0.783	0.814	0.457	0.093, 2.252	0.926	.336
Loyalty	-0.613	0.503	0.542	0.202, 1.452	1.485	.223
Authority	0.745	0.541	2.106	0.730, 6.076	1.898	.168
Sanctity	0.966	0.497	2.627	0.991, 6.961	3.773	.052
Ideological orientation	1.270	0.420	3.560	1.564, 8.105	9.151	.002
Voting intention for the challenging right-wing coalition						
Care	-1.563	1.135	0.210	0.023, 1.937	1.897	.168
Fairness	-0.307	1.096	0.735	0.086, 6.300	0.079	.779
Loyalty	0.327	0.836	1.387	0.270, 7.140	0.153	.695
Authority	-0.605	0.922	0.546	0.090, 3.328	0.431	.512
Sanctity	2.033	0.972	7.638	1.136, 51.337	4.374	.036
Ideological orientation	3.120	0.721	22.641	5.515, 92.940	18.748	< .001
Voting intention for the Movimento Cinque Stelle						
Care	-1.319	0.723	0.267	0.065, 1.102	3.334	.068
Fairness	-0.568	0.837	0.567	0.110, 2.924	0.460	.497
Loyalty	-1.285	0.509	0.277	0.102, 0.749	6.387	.011
Authority	1.025	0.609	2.786	0.845, 9.185	2.833	.092
Sanctity	1.241	0.523	3.460	1.241, 9.642	5.633	.018
Ideological Orientation	0.963	0.430	2.621	1.128, 6.087	5.019	.025

*Note*. Voting intention for the challenger left-wing coalition was the reference group. Ideological orientation: from 1 = left-wing oriented to 5 = right-wing oriented.

As shown in Table 6, ideological orientation was a strong predictor of voting intention. Moreover, consistent with our expectation and with Study 1, Sanctity endorsement predicted participants’ intention to vote for both the right-wing political groups (the Con Monti per l’Italia coalition and the challenging right-wing coalition) rather than for the left-wing coalition. Table 6 also reveals that Sanctity endorsement predicted the intention to vote for the Movimento Cinque Stelle rather than for the left-wing coalition. Moreover, moral concerns for Loyalty predicted the intention to vote for the left-wing coalition rather than for the Movimento Cinque Stelle.

Another nominal regression, where voting intention for the Movimento Cinque Stelle served as the reference group, showed that, always controlling for ideological orientation, Loyalty endorsement predicted also the intention to vote for the challenging right-wing coalition rather than for the Movimento Cinque Stelle (*B* = 1.613, *SE* = 0.790; Wald = 4.167; *OR* = 5.017, 95% CI[1.066, 23.602]; *p* = .041).

**Figure 4:**
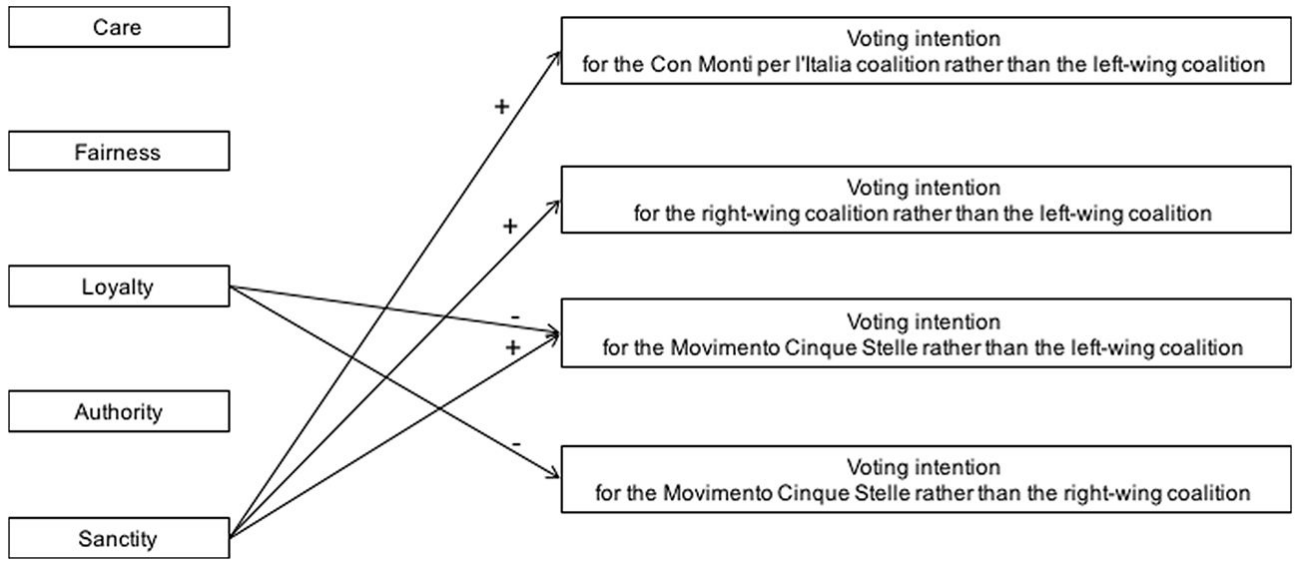
The association between distinct moral foundations and voting intention in 2013 Italian national elections, Study 3. Visualization of the main results obtained in regression analyses, controlling for ideological orientation.

Figure 4 visualizes the association between moral foundations and voting intention, controlling for ideological orientation, as it emerged in Study 3 where the incumbent coalition Con Monti per l’Italia, the two major right-wing and left-wing coalitions, and the Movimento Cinque Stelle were competing in 2013 national elections.

### Discussion

Study 3 confirms that, at real national elections, voting intention can respond to a variety of moral concerns whose influence is not completely accounted for by ideological orientation. Consistent with Study 1 and with previous studies on national elections where a major right-wing candidate and a major left-wing candidate competed (e.g., Franks & Scherr, 2015), Sanctity endorsement was associated with the intention to vote for both the right-wing coalitions rather than for the left-wing one, even when ideological orientation was controlled for.

Moreover, Study 3 shows that, in the peculiar electoral context of the 2013 Italian national elections, Sanctity endorsement also nourished participants’ intention to vote for the Movimento Cinque Stelle rather than for the left-wing coalition. Thus, supporters of the Movimento Cinque Stelle endorsed a category of moral concerns that is typically associated with support for right-wing policy positions and groups. This result uncovers a basic similarity between the Movimento Cinque Stelle and right-wing political groups. Moreover, it was participants’ Loyalty concerns for keeping faithful to one’s political in-group that fostered their intention to support the insiders of the political system, that is, the right- or the left-wing coalitions, rather than an outsider such as the Movimento Cinque Stelle.

## General Discussion

The present research investigates the association between moral foundation endorsement and voting intention in Italy, controlling for the individual’s ideological orientation. Ideology is associated both with vote (Freire, 2006; Van der Eijk et al., 2005) and with moral foundation endorsement (Graham et al., 2009, 2012; Weber & Federico, 2013); thus it could account for the association between moral concerns and vote. However, here we argue that voting intention can respond to moral concerns that the individual’s ideological orientation does not completely capture.

Results obtained showed that as far as the two major right- and left-wing national political groups are concerned, the intention to vote for the right-wing political groups rather than for the left-wing rivals is associated with Sanctity concerns, and that this happens independently from the individual’s ideological orientation. This result is consistent with and extends previous U.S. findings that Sanctity endorsement predicts conservative vote choice (Franks & Scherr, 2015). Thus, the basic moral concern for avoiding contamination and for promoting and preserving physical and spiritual purity is a moral concern that the ideological divide cannot capture but that orient significantly people’s political choices (see also Koleva et al., 2012). This result is also consistent with the finding that people who self-locate in similar ideological positions give different importance to Sanctity (see above Weber & Federico, 2013).

Second, the present research highlights that also moral concerns other than Sanctity can underlie voting intention. Study 1 showed that moral concerns for care of vulnerable people and for respect of established authority turned supporters of both right-wing and left-wing political groups away from voting the autonomist party. Study 2 suggested that moral concerns for equality and justice can be associated with the intention to support extra-parliamentary left-wing political groups rather than the more moderate national left-wing party. Furthermore, Study 2 and Study 3 highlighted that also Loyalty concerns can be associated with voting intention. A closer inspection of the electoral contexts examined in Study 2 and Study 3 suggests that, in each context, this basic moral concern might have taken two specific distinct meanings. In Study 2, moral concerns of Loyalty probably mirrored the perceived righteousness of supporting a (right-wing) candidate deeply based in one’s territorial in-group; in Study 3, moral concerns of being loyal towards one’s political in-group motivated supporters of established (left-wing and right-wing) parties to turn away from a populist movement.

Finally, Study 3 showed that supporters of a political group that on the surface claimed to be an outsider of traditional politics indeed shared the same moral concern for Sanctity that traditional right-wing voters endorse. This result hints at the possibility that moral motivations underlying voting choice may be quite different from the ones that are openly stated (moral dumbfounding; Haidt, 2001). So the investigation of moral foundations associated with vote choice may lead to uncover unexpected similarities (or differences) among supporters of different (or the same) political groups.

Taken together, results obtained suggest that voting choice can be motivated by multiple moral concerns that are not completely accounted for by the individual’s ideological orientation. On the one hand, the examination of moral foundations enables to predict voting intention for political groups that are located on similar sides of the left-right continuum, as well as for extra-parliamentary political groups that distance themselves from traditional political parties and often refuse, at least in words, the left-right dialectic. On the other, it can highlight that voters who seemingly have opposite political positions do share some moral concerns when it comes to complex voting choices. Therefore, extending the examination to fragmented electoral contexts like the Italian ones examined in this research can give us a more complex picture of the relationship between moral foundations and voting choice than the one offered by previous studies carried out in the U.S. bi-polar electoral context.

The present research was limited by the use of convenience and restricted samples. This problem is further exacerbated by the existence of more than two political groups which broke the samples down into small sub-groups, especially in Study 3, where there are four political groups. Thus, although the obtained results are fairly consistent across all studies, this caveat should be considered and future studies involving larger and representative samples are needed. Moreover, the present research investigated voting intention rather than real voting behavior. Franks and Scherr (2015) examined both voting intention and voting behavior at U.S. presidential elections and found consistent results across their studies. Voting intention usually correlates strongly with voting behavior (e.g., Granberg & Holmberg, 1990). This is also the case for Italy. For example, Roccato and Zogmaister (2010) analyzed voting intention and voting behavior at the 2006 national election using a representative sample and found a strong correlation (*phi* = .93, *p* < .001). However, we cannot equate behavioral intention with real behavior and hope that future studies will investigate the association between moral foundation endorsement and vote by analyzing real voting behavior.

Obtained results suggest that political candidates might be advantaged by framing their electoral appeals in terms of moral foundations. Recent studies have shown that when a message elicits moral concerns that are relevant to the audience’s prevailing ones, e.g. a Sanctity-framed pro-environmental message that highlights how contaminated the environment has become and how important it is to clean it, addressed to a right-wing audience, it turns out to be especially persuasive (Feinberg & Willer, 2013). Message frames based upon moral foundations can partly shift the audience’s political attitudes, mainly by reinforcing them, if they are based on the foundations that the audience is likely to endorse (Day, Fiske, Downing, & Trail, 2014). Future experimental studies could investigate whether candidates’ electoral appeals that are framed in terms of relevant moral foundations can affect also participants’ intention to vote for them. As compared with unframed appeals, morally framed appeals could strengthen participants’ voting intentions by adding a sense of moral obligation to vote.

Overall, our investigation of the multiple categories of moral concerns that can be associated with vote in fragmented and complex electoral contexts suggest that voters can charge their choice with a range of moral concerns that do not descend from their ideological orientation only. Rather, the comparative context in which voters have to make their choice, i.e. the simultaneous consideration both of the supported candidate and of his/her competitors, can contribute significantly to charge the voters’ choice with specific moral concerns. Our results suggest that the comparative context of each election can contribute to determine which moral foundation would be more effectively elicited in order to foster the intention to vote for a given candidate or political group. Not only should political candidates consider the categories of moral concerns that are typically associated with the ideological position of their political group; they should also take into account and leverage the categories of moral concerns that their competitors are likely to elicit in the electorate.

To conclude, this research shows that voting choice can respond to a range of moral concerns in a way that is not completely accounted for by the individual’s ideological orientation. Studying the association between vote and the individual’s moral concerns based on the pluralistic view of morality proposed by the Moral Foundation Theory in fragmented and not perfectly bipolar electoral contexts has the potential to shed light on this issue and to offer a multifaceted view of the various meanings voters can associate to their choice.
